# Chemical biology tools for protein labelling: insights into cell–cell communication

**DOI:** 10.1042/BCJ20220309

**Published:** 2023-09-21

**Authors:** Megan H. Wright

**Affiliations:** School of Chemistry and Astbury Centre for Structural Molecular Biology, University of Leeds, Leeds LS2 9JT, U.K.

**Keywords:** biochemical techniques and resources, cell–cell communication, chemical biology, proteomics, signalling

## Abstract

Multicellular organisms require carefully orchestrated communication between and within cell types and tissues, and many unicellular organisms also sense their context and environment, sometimes coordinating their responses. This review highlights contributions from chemical biology in discovering and probing mechanisms of cell–cell communication. We focus on chemical tools for labelling proteins in a cellular context and how these can be applied to decipher the target receptor of a signalling molecule, label a receptor of interest *in situ* to understand its biology, provide a read-out of protein activity or interactions in downstream signalling pathways, or discover protein–protein interactions across cell–cell interfaces.

## Introduction

Cell–cell communication is ubiquitous in biology with fundamental roles in organismal development, homeostasis, growth, and response to the environment and pathogens. A common mode of communication involves the release of a biochemical signal (small molecule, peptide, or biomacromolecule such as a protein) by one cell that is detected by a receiving cell. Cells can also directly contact one another through biomolecular interactions e.g. between proteins and/or other biomolecules such as glycans, and interaction can also result in the translocation of material from one cell to another. For example, there are a predicted 2200 human genes that encode secreted proteins, another ∼2000 encoding proteins with extracellular domains that presumably sense signals [1], and an unknown number of small molecule primary and secondary metabolites with the potential to act as signalling molecules. Add to this the intracellular receptors that detect lipophilic molecules, such as the nuclear receptors, the potential for hundreds of proteins to be regulated by more promiscuous signals like reactive oxygen species (ROS), and the ability of cells to tailor signalling (e.g. via degradation of signalling molecules), and it is clear that a significant proportion of the cellular machinery is dedicated to transmitting, sensing and responding to extracellular stimuli. Basic questions to address in investigating a cell–cell communication mechanism include: What is the signal? How is the signal detected? How is the message relayed? What is the cellular response? More complex questions might be: What cross-talk exists in this system? What feedback loops are operating? How are multiple incoming signals integrated? Understanding the mechanistic basis of cellular communication is critical to understanding its role in health and disease, and enabling it to be modulated, for example to treat disease.

Molecular biology and genetic approaches have over the years defined many molecular mechanisms involved in cell–cell communication but many signals and receptors remain orphan and many may remain undiscovered. In a multi-cell type or multi-species system it can be difficult to obtain clear answers using genetic approaches, especially where a single signal or receptor is involved in a multitude of biological pathways, as cells can often adapt and mitigate the effects of knock-out or knock-down. Chemical approaches can augment and complement molecular biology, typically by using small molecule tools that can act in a rapid fashion to analyse or modulate a biological system.

**Figure 1.**
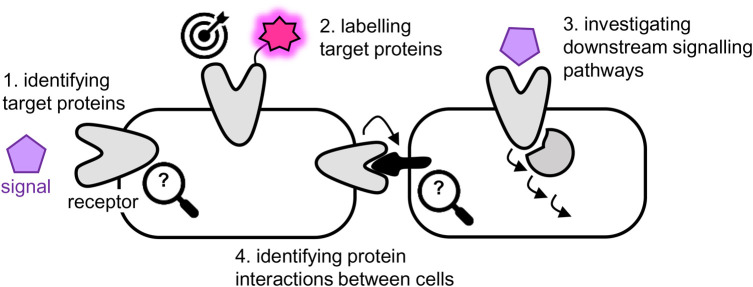
Chemical biology approaches for cell–cell signalling covered in this review.

Chemical biology approaches to cell–cell communication are broad and include: synthetic biology approaches that range from installing reporters or sensors in a subset of cells in a population, to building whole new synthetic systems [2–4]; modulators that perturb a signalling pathway e.g. chemical probes, caged and switchable signals or responders, bump-hole approaches; and labelling tools that give a read-out (e.g. fluorescence) of the presence/activity of signals, receptors or downstream pathways. Here, we focus on chemical approaches to label proteins in living cells in order to define molecular interactions operating during cell–cell communication and to help answer the questions of how signals are detected and how messages are relayed (Figure 1). First we describe the use of chemical tools that mimic signalling molecules to trap interacting protein partners in cells, aiding target and mode-of-action deconvolution. Next, we discuss tools developed to label specific proteins, focusing on G-protein-coupled receptors as an example, and tools to read-out phosphorylation in pathways downstream of signal engagement. Finally, we cover proximity mapping methods to identify protein–protein interactions at cell–cell interfaces.

## Identifying the protein targets of signals

**Figure 2.**
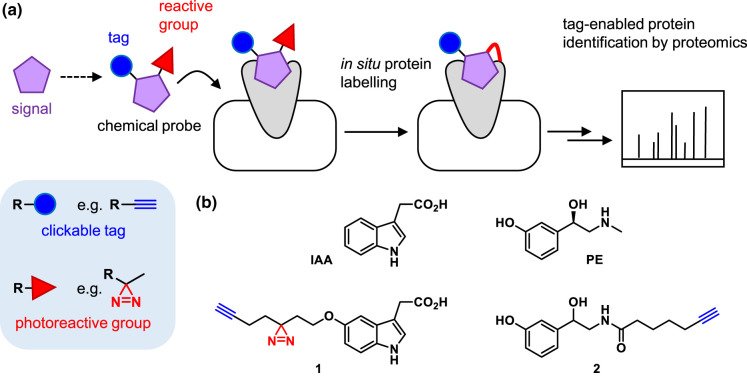
Photoaffinity labelling for identifying the protein targets of signalling molecules. (**a**) Overview of the approach. (**b**) Example signals (top) and their corresponding photoaffinity probe (bottom, **1** and **2**) that have been applied to study host-microbe signalling. Alkyne clickable tag — blue; photoreactive group — red.

Chemical proteomic approaches for identifying the targets of bioactive small molecules, which are becoming well-embedded in drug discovery pipelines [5], can be applied to match a signalling molecule with unknown target to its receptor. Here, a labelling probe is created that retains the bioactivity of the parent signalling molecule but is equipped with additional functionality, such as a label (fluorophore, affinity handle, clickable tag) and a reactive group (photoreactive or intrinsically reactive, Table 1). The reactive group generates a stable covalent link between the probe and target proteins, enabling their detection or identification via the label (Figure 2a). This molecule-centric approach works best when a molecule is tractable to synthetic chemistry and where there are structure-activity relationships and assays to guide design. Chemical proteomics has been applied to microbial metabolites that are sensed by host organisms [6,7], to the detection of host bile acids by enteric bacteria [8], and to deciphering targets of interspecies signalling [9–15], where signalling molecules from one species are detected by another. Two recent case studies in host-microbe interactions are described here as examples of this type of approach.

**Table 1:** Summary of commonly used tags and reactive groups for chemical tool-based protein labelling in cells referred to in this review

Name	Structure	Application notes
**Analysis tags**		
Alkyne	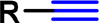	Minimal bioorthogonal tag. Can be reacted via a copper-catalysed click reaction. Due to cellular toxicity of copper this tag is normally reacted in lysate.
Azide	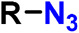	Minimal bioorthogonal tag. Can be reacted with terminal alkynes (in copper-click reactions) or strained alkynes (copper-free) for cellular applications.
Biotin	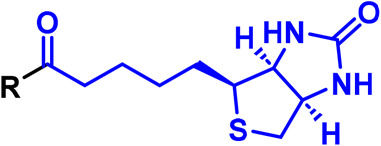	Has high affinity to the protein Streptavidin/neutravidin. Used in affinity enrichment on solid support (Streptavidin-coated resin).
Desthiobiotin	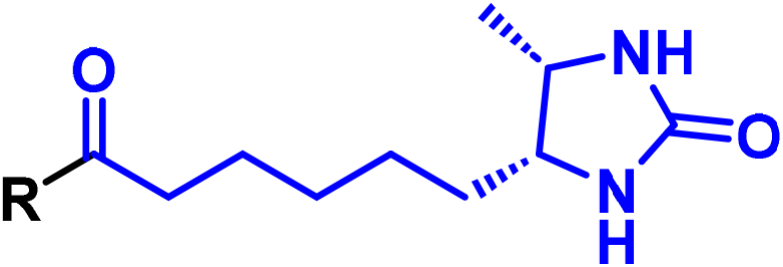	Used in affinity enrichment on Streptavidin resin. Lower affinity than biotin facilitates elution from resin.
Fluorophore e.g. TAMRA	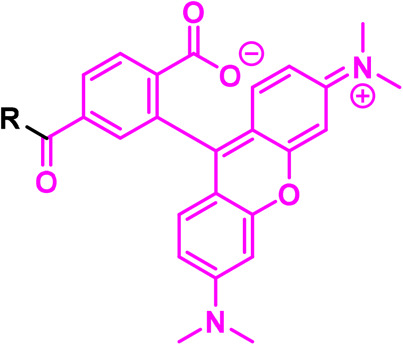	Many fluorophores are available, here TAMRA is provided as an example. Used for cellular imaging by microscopy and for visualisation of labelled proteins on SDS–PAGE gel.
**Photoreactive groups**		
Alkyl diazirine	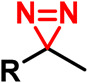	Activated by irradiation with UV light (∼365 nm), leading to loss of N_2_ and generation of a carbene, which rapidly inserts into adjacent X-H bonds.
Trifluoromethyl aryl diazirine	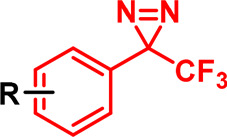	Activated by irradiation with UV light (∼365 nm), leading to loss of N_2_ and generation of a carbene, which rapidly inserts into adjacent X-H bonds.
2,5-tetrazole	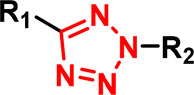	Activated by irradiation with UV light (∼305 nm), leading to formation of a nitrile imine intermediate that reacts with nucleophiles, especially carboxylic acids (Glu and Asp).
**Intrinsically reactive groups/linkers**		
Sulfonyl fluoride	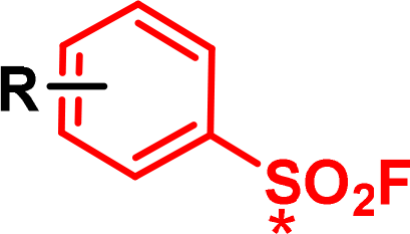	Intrinsically reactive via SuFEx chemistry at S (*). Can react with Lys, Tyr, Cys, Ser, His, Thr, depending on context. Tunable via substituents on ring.
Acyl imidazole	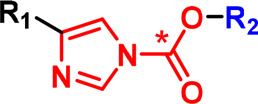	Reactive linker used in LDL. Protein nucleophiles (e.g. Lys) attack the carbonyl with loss of imidazole unit and transfer of -CO_2_R_2_ to the protein surface.
Phenyl ester	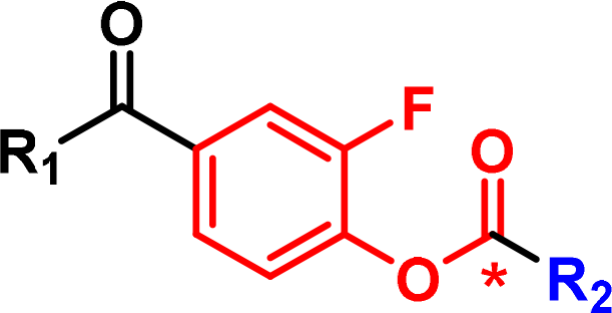	Reactive linker used in LDL. Protein nucleophiles (e.g. Lys) attack the carbonyl with loss of the aromatic ring and transfer of -COR_2_ to the protein surface.
NASA	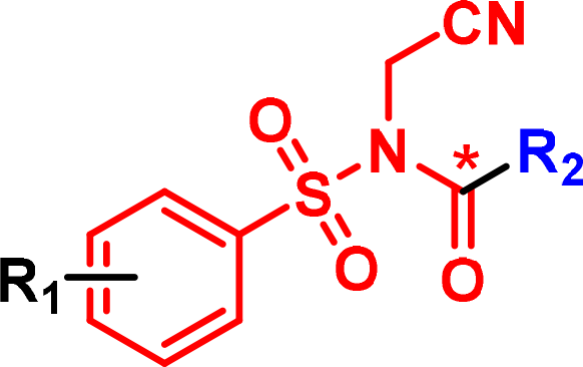	Reactive linker used in LDL. Protein nucleophiles (e.g. Lys) attack the carbonyl with loss of the activated acyl sufonamide and transfer of -COR_2_ to the protein surface.

LDL, ligand-directed labelling.

A recent study used a chemical probe-based approach to define the human protein targets of microbe-derived metabolites such as indole-3-acetic acid (IAA, Figure 2b), which are implicated in host signalling pathways [16]. Photoaffinity probe **1** was designed as a close structural mimic IAA but was equipped with a diazirine photoreactive group and alkyne clickable tag. This probe was applied in a human colorectal cancer cell line to identify protein targets via a chemical proteomic workflow: following probe incubation with the cells and UV irradiation to crosslink to target proteins, cells were lysed and a click reaction performed to attach biotin to probe-crosslinked proteins, which were then enriched via neutravidin resin and identified by mass spectrometry-based proteomics. A particularly interesting hit was GPRC5A, an orphan GPCR with no previously known ligands. The authors further showed that microbial monoamine indole metabolites activated GPRC5A and identified specific microbes producing these. They linked agonism of GPRC5A to immune and cancer signalling pathways.

The second exemplar study demonstrated the interaction of human catecholamine hormones with the chemotaxis coupling protein CheW in the marine bacterium *Vibrio campbellii* [17]. Catecholamines trigger various responses in *V. campbellii* and other bacteria, promoting virulence, biofilm formation, motility, and iron uptake. A probe based on phenylephrine (PE, Figure 2b), a synthetic agonist of human α-adrenoreceptors, was designed and found to promote bacterial growth but not iron uptake. This was critical in enabling the authors to deconvolute the polypharmacology of catecholamines: some of these chelate iron but by selecting a compound without this feature, the authors could focus on identifying proteins driving the other phenotypes. Probe **2** incorporated a click tag and was intrinsically photoreactive. Through a series of chemical proteomic experiments, CheW was identified as the bacterial target of phenylephrine and was validated in follow-up assays.

These examples illustrate both the strengths and weaknesses of chemical proteomic approaches. In both studies, careful control proteomic experiments had to be performed to tease out the potential true hits from a background of proteins that bound the probes. On the other hand, it is hard to imagine another experimental tool that could profile an entire proteome for potential targets in such an unbiased manner. Outputs also require careful follow-up to validate the functional relevance of hits, since not all interactions will be important for bioactivity. Where genetic resources exist and there is a clear hypothesis for which receptor class might be involved, more traditional approaches might be just as effective. For example, proteins involved in the response to autoinducer-2 (AI-2), a widespread bacterial quorum sensing molecule, were identified in *P. aeruginosa* by analysing chemoreceptor mutants [18]. This approach provides a more direct link to function (since the protein is implicated in the mechanism of response) but direct binding to the signalling molecule is inferred, not shown. Thus a strength of photoaffinity labelling is in demonstrating direct target engagement in a cellular context.

Another challenge with photoaffinity-based approaches is to design a probe that recapitulates the bioactivity of the parent compound, and gives rise to the same phenotypes or perturbation of downstream pathways. Good structural activity relationships and, sometimes, significant synthetic effort is required. The larger the signalling molecule, the less problematic incorporation of unnatural functionality is likely to be. However, if the photoreactive group is far from the binding interface, then the complex will not be trapped. Thus it is a careful balancing act to position a photoreactive group where it will be able to crosslink but will not prevent binding. For proteins as signalling molecules, Tian et al. recently reported a suite of approaches where secreted proteins are labelled with multifunctional reagents containing both a reactive group and biotin for enrichment [19]. Long linkers were used to ensure that targets could still be captured. To enhance sensitivity, the authors made use of glycans for labelling the signal and for crosslinking to the receptor. This method enabled discovery of a new protein–protein interaction involved in pancreatic cancer signalling.

## Ligand-based labelling tools for receptor or sensor proteins: GPCRs as a case study

Once an interesting receptor or sensor protein has been identified, various molecular biology or chemical tools can be developed to further elucidate its biology. Despite the wealth of information available from genomics, most biomedical research focuses on a small percentage of human proteins [20]. However, it is well known that the availability of a pharmacological modulator (usually a small molecule) can transform research into that target protein [20]. For G-protein-coupled receptors (GPCRs), a family of human proteins involved in signal transduction and the largest class of drug targets [21], there is a long history of using ligand-based reagents, typically modified with radioactive or fluorescent labels, to develop assays and study receptor biology [22]. Covalently attaching a reagent to a receptor can offer advantages, such as facilitating detection or studies of receptor trafficking. Here, we highlight this class of reagent, where ligands are engineered to contain either a photoreactive or an intrinsically reactive group that enables covalent attachment of the reagent, or part of the reagent, to the receptor in cells.

**Figure 3.**
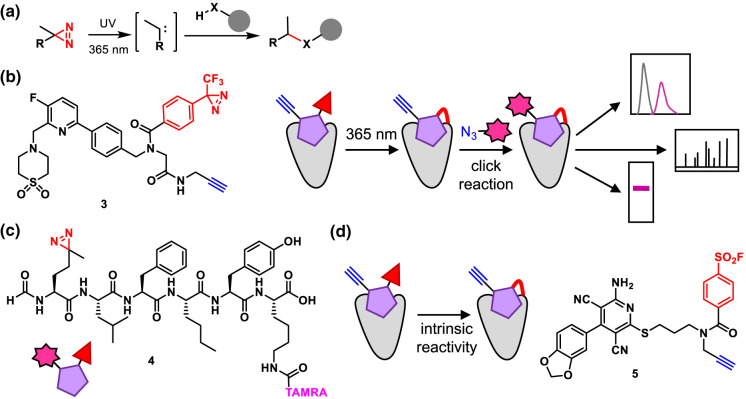
Chemical probe-based labelling of receptors involved in cellular signalling. (**a**) Photoactivation of a diazirine and subsequent reaction of the carbene intermediate with a protein target. (**b**) Small molecule photoaffinity probe **3** applied for CB2R labelling and downstream analysis via flow cytometry, mass spectrometry and SDS–PAGE following click reaction to introduce a label (fluorophore or biotin). (**c**) Peptidic photoaffinity probe **4** for FPR1 labelling. (**d**) Electrophilic probe **5** for modification of A_1_AR.

Photoaffinity labelling probes like those discussed above contain a photoreactive group incorporated into the ligand and triggered by light to form a reactive species that crosslinks with residues on the protein. Benzophenones and aryl azides have been used historically but diazirines have become the photoreactive group of choice in recent years due to their fast reaction that has been hypothesised to minimise non-specific background labelling, and small size (Figure 3a) [23]. They are efficiently activated by 365 nm UV light which is not overly damaging to biological systems. Disadvantages are alternative photoactivation pathways leading to longer-lived diazo species that bias reactivity towards acidic residues, particularly in the case of alkyl diazirines, and low photolabeling yields [24].

Recent examples illustrate some of the potential capabilities and challenges with these reagents. Soethoudt et al. [25] designed a photoaffinity probe (**3**, Figure 3b) for the cannabinoid CB_2_ receptor based on a small molecule agonist and incorporating a diazirine and an alkyne tag for click attachment of a fluorophore or biotin. After validation in overexpression systems, the authors showed that the probe could be used to detect endogenous CB_2_R in primary human immune cells using fluorescence-activated cell sorting. With any fluorescent ligand, background or off-target, labelling needs to be carefully assessed. Covalent ligands can help in evaluating this. A chemical proteomics workflow involving attachment of biotin to probe-labelled proteins, enrichment and mass spectrometry-based proteomics identified not only CB_2_ as a target of probe **3** but also five other proteins that may be off-targets of the parent compound. Similarly, metabotropic glutamate receptor 2 (mGlu2) photoaffinity probes localised in cellular membranes as well as labelling the receptor [26]. Diazirines can also be readily incorporated into peptidic ligands. A recent example is our report of a formylpeptide-based photoaffinity probe for formylpeptide receptor 1 (FPR1), **4** (Figure 3c), which enabled imaging of FPR1, assessment of the off-targets of the peptides in cells, and a means for evaluating the on-target activity of FPR1-directed ligands [27].

Beyond photocrosslinkers, intrinsically electrophilic groups can be incorporated into ligands; these react spontaneously when positioned close to nucleophilic protein side chains. Sulfonyl fluorides are popular due to their ability to label a broad range of residues (Lys, Tyr, His, Cys, Ser) [28]. A recent study applied a sulfonyl fluoride in a probe, **5** (Figure 3d), targeting the adenosine A_1_ receptor (A_1_AR) [29]. Chemical proteomic analysis to identify the proteins labelled by the probe found excellent signal to noise when using overexpression systems, but the presence of off-targets in samples where A_1_AR was expressed at endogenous levels. As noted by the authors, this highlights the challenge of detecting very low levels of native GPCRs with covalent probes.

**Figure 4.**
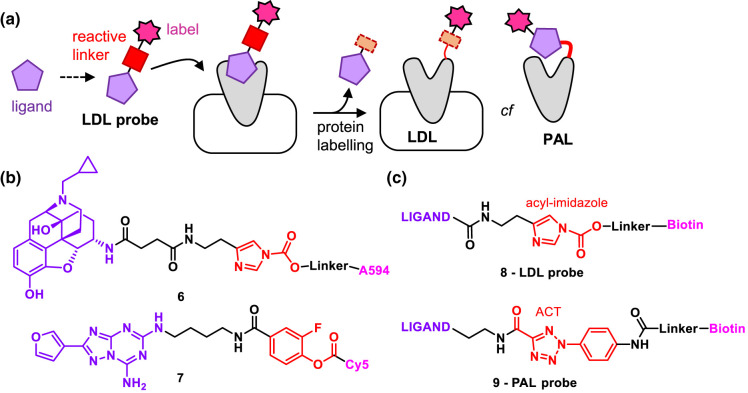
Ligand-directed labelling (LDL) chemistry applied to receptors involved in cell signalling. (**a**) Concept of LDL compared with PAL (photoaffinity labelling). (**b**) Example LDL probes that have been applied for labelling of opioid receptors (**6**) and A_2A_R (**7**). (**c**) Two probes, **8** and **9**, from a study comparing various PAL and LDL strategies for DRD2.

An evolution of covalent labelling is ligand-directed labelling (LDL), where a reactive group is incorporated into a ligand but takes the form of a labile linker. When brought close to protein nucleophilic side chains by reversible binding, the linker is cleaved, resulting in transfer of a label (e.g. a fluorophore, biotin or click tag) onto the protein surface and leaving the ligand free to diffuse away (Figure 4a) [30]. Pioneered by the Hamachi group, a key advantage of this approach is that, after washing away the ligand, the protein may be able to resume its normal biological functions. Thus LDL is a potential method to label a protein in any sample at endogenous levels with a small fluorophore without the need for genetic engineering. In the case of proteins involved in cell–cell communication, this method can label a native protein for downstream analysis of its signalling behaviour in response to stimuli. Miki et al. [31] used LDL to transfer labels onto membrane-bound carbonic anhydrase 12, bradykinin B_2_ receptor, N-methyl-d-aspartate (NMDA) receptor, and Folate receptor, enabling pulse-chase analysis of their half-lives and degradation pathways in cells. LDL has also been applied to opioid receptors, including for imaging receptors in living brain tissue (probe **6**, Figure 4b) [32]. Finally, a LDL reagent was used to label the adenosine A_2A_ receptor, allowing detection of this receptor at endogenous levels (probe **7**) [33].

Currently there are few comparative studies of covalent reagents and it remains to be seen which technology (photoaffinity, electrophilic or LDL) is most appropriate in which circumstances. One recent study applied arylcarboxytetrazoles (ACTs) [34] as photoreactive groups in probes for covalent labelling of Human Dopamine Receptor D2 (DRD2), and also compared these with other photoaffinity (diazirine, aryl azide and benzophenone) and LDL probes [35]. Interestingly, the alkyl diazirine probe and ACT-containing probe (probe **9**, Figure 4c) significantly outperformed probes containing an aryl azide, benzophenone, or acyl imidazole (probe **8**). This may reflect the fact that alkyl diazirines [24] and ACTs [34] preferentially react with acidic residues, which are abundant on membrane protein extracellular surfaces. This suggests that optimal positioning of probe and protein reactive groups may be key to achieving efficient labelling. All these approaches are greatly facilitated by structural information to guide design and placement of reactive groups; this is particularly the case where the reactive group has narrow specificity in terms of the protein residues it will react with.

Further comparative studies are really needed to understand how best to design these protein-centric labelling tools. The emergence of further diverse covalent and reactive chemistries, structural information on ligand recognition, high potency ligands, and high-throughput approaches to make probe discovery more efficient, should also help drive the development of these technologies. Here, we have focused on GPCRs as there is growing activity in this area; whilst these are clearly important proteins for cellular communication, there are many other protein families where similar tools could be more widely applied.

## Chemical tools to read-out downstream signalling events: phosphorylation as a case study

The chemical tools described in the two sections above bind, usually functionally, to a receptor by mimicking an endogenous ligand or pharmacological agent. However, ligand binding is just the start of signalling cascades that cause alterations in cellular behaviour, and chemical biology approaches have also been developed to address events downstream of ligand-receptor binding. Due to its central role in signal transduction pathways, here we highlight a few examples of tools to study protein phosphorylation, both in eukaryotes and prokaryotes.

**Figure 5.**
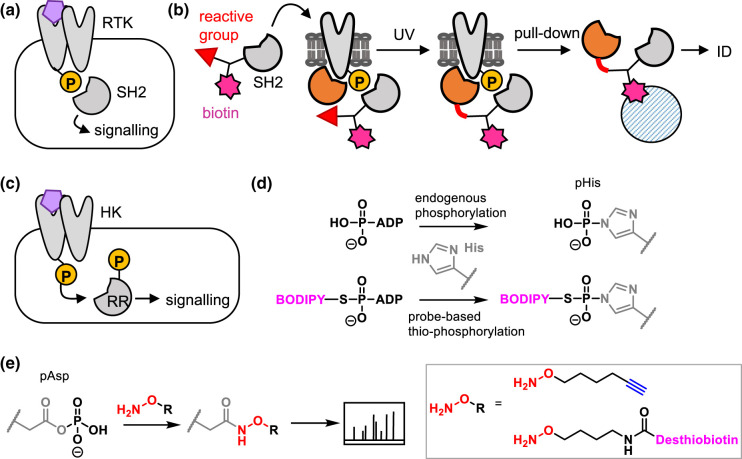
Chemical tools for signalling pathways downstream of receptor activation that involve protein phosphorylation. (**a**) Receptor tyrosine kinase (RTK) signalling via phosphorylation and recognition of phosphorylation site by SH2 domains. (**b**) Application of a SH2-based chemical tools for trapping interactors of RTKs. (**c**) TCS (two component system) signalling in bacteria, consisting of HK (histidine kinase) and RR (response regulator) proteins that relay signalling via transient phosphorylations. (**d**) Application of an ATP-based probe for labelling active HKs via the pHis. (**e**) Detection of phospho-aspartate (pAsp) using hydroyxylamine-based probes.

Phosophotyrosine (pTyr) signalling is important in cell–cell signalling in multicellular organisms. It is frequently involved in control of proliferation, differentiation and migration, and its dysregulation is linked to cancer. Receptor tyrosine kinases (RTKs) receive extracellular signals, typically promoting receptor dimerisation and resulting in auto-phosphorylation of tyrosine residues on intracellular domains. These pTyr residues are recognised by intracellular ‘reader' proteins, in particular those containing Src homology 2 (SH2) domains, triggering a signalling cascade (Figure 5a). Interactions between the reader and receptor proteins are often transient and difficult to detect by traditional pull-down approaches. The Tian group developed an approach in which engineered SH2 domains were functionalised with biotin and a photoreactive group, then added to lysates where they bound to pTyr-containing proteins (Figure 5b) [36]. UV irradiation trapped proximal interacting proteins and the biotin tag enabled pull-down and proteomic identification of interactors. The researchers showed that their method was applicable in clinical samples and used it to discover that the RTK PDGFRB was functionally important in cell–cell signalling in tumours.

In bacteria, extracellular signals also trigger phosphorylation-dependent signalling pathways. Histidine kinases (HKs) are membrane proteins that autophosphorylate on histidine when stimulated by a signal (Figure 5c). The phosphate group is then transferred to an aspartate residue on a response regulator (RR), which goes on to regulate gene expression. The HK and RR form a two component system (TCS). However, these phosphorylations pose a significant analytical challenge: neither pHis (phospho-histidine) nor pAsp (phospho-aspartate) easily survive standard proteomic workflows, hindering assessment of TCS activity. Historically proteins were studied using low-throughput approaches such as radiolabelled ATP and gel analysis. Several chemical biology approaches address these challenges. The Carlson group developed a suite of ATP analogues as probes to label active HKs (Figure 5d) [37,38]. These take advantage of the improved stability of a thio-pHis intermediate to enable stable attachment of a fluorophore to the HK. Although they are not cell permeable these probes have enabled screens for HK inhibitors [39].

The ATP-based probes show some transfer to the pAsp of the RR [37] but probes have also been developed to directly detect this labile modification [40,41]. Hydroxylamine-based probes functionalised with desthiobiotin for pull-down or a clickable alkyne directly labelled pAsp residues in native proteomes, each enriching hundreds of pAsp sites in bacterial proteomes (Figure 5e).

These examples showcase the strength of chemical biology approaches that apply covalent chemical tools to trap transient molecular interactions or protein modifications that are challenging to detect by more traditional biochemical methods. Here we have focused on phosphorylation events resulting from signal:receptor interactions, but a much wider array of post-translational modifications (PTMs) are involved in cellular signal transduction and integration. For example, ubiquitination marks receptors such as EGFR for degradation following internalisation [42], and *O*-GlcNAcylation is implicated in response to insulin [43] and in neurotransmission [44]. There is signficant work in developing chemical tools to investigate ubiquitin signalling [45] and *O*-GlcNAcylation [46] that could be applied to study questions in cell–cell communication.

## Proximity labelling to detect cell–cell interactions

**Figure 6.**
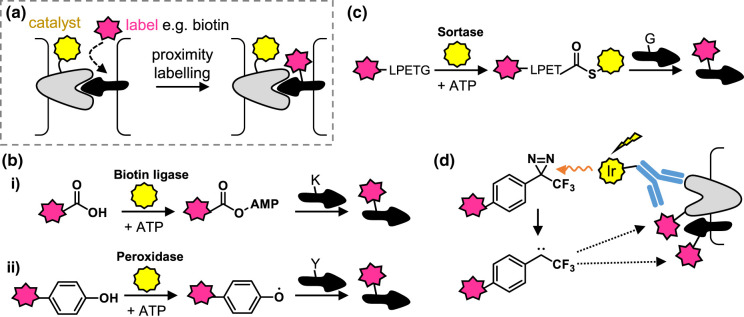
Proximity labelling approaches for protein-protein interactions between two cells. (**a**) Concept of proximity labelling for discovering protein–protein interactions at cell–cell interfaces. (**b**) Two widely applied enzyme-based proximity labelling approaches using (i) a ligase (e.g. BioID) and (ii) a peroxidase (e.g. APEX). Reactive intermediates are generated that label proteins in close proximity. (**c**) Contact-dependent enzyme-based proximity labelling approach EXCELL which generates a covalent enzyme intermediate that is able to label interacting proteins. (**d**) MicroMap approach that uses an iridum photocatalyst to activate diazirines towards protein labelling. Here, the photocatalyst is targeted to the receptor of interest via conjugation with an antibody.

As well as informing on receptor-signal interactions and the pathways that they trigger, chemical biology approaches are also useful for determining which proteins are in close proximity and thus potentially interacting, for example across cell–cell interfaces. A suite of established and emerging proximity labelling methods come in two main flavours: enzyme-mediated and photocatalytic [47]. In both cases, a reagent (biochemical or chemical) is directed to a specific cellular location, say a protein on the cell surface, where it typically activates *in situ* another, soluble, reagent to generate a reactive species that labels proteins in the vicinity (Figure 6a). Often the soluble reagent incorporates biotin for downstream enrichment and identification of proteins via proteomics, or for visualisation via immunostaining.

Enzyme-based systems (Figure 6b) include engineered biotin ligases that generate protein-reactive active esters (e.g. BioID [48], TurboID [49]); peroxidases that generate radicals (e.g. HRP [50], APEX [51]); and protein-based photosensitisers that generate singlet oxygen when irradiated (e.g. miniSOG [52]), oxidising proteins that then react with biotin-functionalised amine/thiol reagents. These approaches vary in their temporal and spatial resolution, related to the lifetime of the reactive species and how far it diffuses from the site of activation before reacting. BioID requires labelling over hours, whereas this is reduced to minutes for TurboID [49]. The temporal resolution of TurboID can be further improved by using a variant where the catalytic lysine is caged, rendering the enzyme inactive until application of UV light [53]. The lifetime of the radical in peroxidase-based approaches is <1 ms, leading to a radius of labelling that has been measured at 200–300 nm for cell surface labelling [50,54], although this will presumably vary in different environments according to molecular crowding and the local concentration of radical quenchers. TurboID has been applied to map proteins at epithelial cell junctions, through fusion of the enzyme to the extracellular domain of E-cadherin [55], and APEX has been applied at synapses [56].

Another set of approaches, termed ‘contact-dependent' [57], exploit site specifically introduced enzymes that transfer substrates (peptides, proteins or glycans) onto neighbouring proteins. Here the radius of labelling is tight because the enzyme is required to bind both the target biomolecule and the reagent for transfer i.e. the activated species does not diffuse from the enzyme. LIPSTIC, ‘Labelling Immune Partnerships by SorTagging Intercellular Contacts’, for example, uses sortase fused to a protein on a donating cell to transfer a label to a receiving cell engineered to express a polyglycine (G5)-tagged protein on its surface [58]. Building on this work, Ge et al. [59] evolved a more promiscuous sortase variant that transfers labels to monoglycine on proteins, which are sufficiently common to avoid the need to genetically engineer the receiving as well as donating cell. They applied this approach, termed enzyme-mediated proximity cell labelling (EXCELL, Figure 6c), to the CD40-CD40L interaction. PUP-IT, which uses a ligase fused to a protein of interest to transfer a small protein Pup-E to neighbouring proteins [60], and Fuco-ID [61,62], which uses a fucosyltransferase to modify cell surface sugars with fucose-biotin, are other contact-dependent methods that have been applied to cell–cell interactions, again in immune signalling. Proximity labelling has also been applied to examine intracellular signalling cascades, for example to monitor *O*-GlcNAc modification of proteins in response to insulin [63].

The second class of proximity-labelled methods uses chemical rather than enzyme catalysts. MicroMap uses an iridium photocatalyst targeted to a location on the cell surface to activate a soluble diazirine reagent to label proximal proteins [64] (Figure 6d). The catalyst is selectively excited by visible light, and nearby diazirines are then excited via Dexter energy transfer from the catalyst, leading to their decomposition into carbenes that can readily insert into biomolecules or are rapidly quenched in water. The short lifetime of the carbene limits the radius of labelling, which is tunable by switching out the diazirine reagent for other photoactivatable groups [54]. MicroMap was used to investigate proteins at the T cell/antigen-presenting cell immunosynapse, by conjugating the catalyst to antibodies against two antigens (PD-L1 or CD45). Two further examples that exploit catalyst-labelling reagent systems for cell–cell interactions are PhoTag (which uses a flavin-based cofactor photocatalyst to generate phenoxy radicals that label protein tyrosine residues) [65] and PhoXCell (which uses dibromofluorescein to generate singlet oxygen, oxidising nearby proteins that can then be labelled by soluble amine-based reagents) [66].

The field of photocatalytic tagging is rapidly expanding and lends itself well to profiling interactions between native cells, at least where a well-characterised antibody exists to allow the photocatalyst to be targeted. Applications of these approaches to cell–cell communication are still scarce but if matched with the appropriate model question these methods could have significant impact. MicroMap has recently also been expanded to small molecule target identification: an iridium catalyst attached to a bromodomain ligand was used in live cells together with a diazirine-biotin reagent to show labelling of the ligand's expected targets [67]. This could provide an alternative to photoaffinity labelling (discussed above) for identifying the targets of small molecule signals.

## Conclusions

There are a wide variety of chemical biology tools that can be used to investigate cell–cell communication. Here we have highlighted reactive reagents to discover the receptors of signals in an unbiased way, and those designed to label receptors of interest to study their biology. We have also discussed chemical tools to investigate downstream signalling pathways, and proximity-labelling approaches to define molecular interactions occurring at cell–cell interfaces. These approaches share common ground in exploiting chemical reactivity to enable specific protein labelling in a cellular context. Strengths of chemical biology approaches include: enabling studies in cells, often on native cells or tissue; temporal control; and data-driven unbiased discovery, usually facilitated by proteomics, itself a rapidly evolving technology. Alternatively, genetic tools to introduce tags and unnatural functionality, or to fuse enzymes (e.g. in proximity labelling approaches), can be combined with chemical tools in powerful ways to provide insight into molecular interactions in cells.

Other areas of cell–cell communication that this review has not discussed include approaches to study cell surface glycans that are intricately involved in interactions between cells and with pathogens. Metabolic Oligosaccharide Engineering to introduce unnatural tags (such as click tags, photoreactive groups) into glycans [68] is well established and a recent study developed an approach for cell-specific tagging, enabling the study of glycoproteomes in co-culture systems and in *in vivo* tumour models [69]. Protein tagging, via a clickable methionine analogue that is metabolically incorporated into newly synthesised proteins, has also been used to monitor secreted proteins and how these change over time in response to lipopolysaccharide treatment of cells [70]. There is also scope for application of activity-based probes, chemical tools that label active enzymes, to study cell–cell interactions: for example probes were used to demonstrate secretion of active KLK proteases into the extracellular space by prostate cancer cell lines when stimulated by osteoblasts, implicating these enzymes in prostate cancer metastasis to bone [71].

While chemical biology technologies continue to evolve, there is also increasing appetite for applying these in more complex systems and with native expression levels. This is a challenge for small molecule ligands, which can be promiscuous binders and must be used at appropriate concentrations to avoid off-target effects [72,73], but these reagents offer the opportunity to investigate clinically relevant samples that are not tractable to genetic manipulation, as several studies highlighted above have begun to do.

Finally, there is an opportunity for applying existing chemical tools (and developing new ones) to address other aspects of signalling. For example, a growing set of chemical tools allows profiling of transient redox modifications on proteins in cells [74]. Reactive oxygen species such as H_2_O_2_ that affect redox PTMs are implicated in cell–cell communication (e.g. [75]) but only a few studies (e.g. [76]) have so far applied chemical biology approaches to such questions. Redox signalling and other emerging phenomena in cell–cell communication will surely benefit from the rapidly expanding toolbox of chemical biology technologies for protein labelling.

## Open Access

Open access for this article was enabled by the participation of University of Leeds in an all-inclusive *Read & Publish* agreement with Portland Press and the Biochemical Society under a transformative agreement with JISC.

## Abbreviations

A_1_AR: A_1_ receptor
ACTs: arylcarboxytetrazoles
DRD2: dopamine receptor D2
FPR1: formylpeptide receptor 1
GPCRs: G-protein coupled receptors
HK: histidine kinase
IAA: indole-3-acetic acid
LDL: ligand-directed labelling
PAL: photoaffinity labelling
PTMs: post-translational modifications
RR: response regulator
RTK: receptor tyrosine kinase
SH2: Src homology 2
TCS: two component system
